# The boron uptake via LAT1 and survival analysis after boron neutron capture therapy in canine hemangiosarcoma cell lines

**DOI:** 10.3389/fvets.2026.1763965

**Published:** 2026-05-12

**Authors:** Ryota Iwasaki, Sho Uchida, Ryutaro Yoshikawa, Sachiko Yoshihashi, Minoru Suzuki, Takashi Mori

**Affiliations:** 1 Department of Veterinary Medicine, Obihiro University of Agriculture and Veterinary Medicine, Obihiro-shi, Hokkaido, Japan; 2 KyotoAR Advanced Veterinary Medical Center, Kyoto, Japan; 3 Joint Department of Veterinary Medicine, Faculty of Applied Biological Sciences, Gifu University, Gifu-shi, Gifu, Japan; 4 Animal Medical Center, Faculty of Applied Biological Sciences, Gifu University, Gifu-shi, Gifu, Japan; 5 Department of Applied Energy, Graduate School of Engineering, Nagoya University, Nagoya-shi, Aichi, Japan; 6 Institute for Integrated Radiation and Nuclear Science, Kyoto University, Sennan-gun, Osaka, Japan

**Keywords:** BNCT, BPA, dog, HSA, L-type amino acid transporter 1, reactor

## Abstract

**Introduction:**

Canine hemangiosarcoma (HSA) is a highly aggressive malignancy with limited effective therapies. Boron neutron capture therapy (BNCT) has begun to be recognized as a potential treatment in human angiosarcoma, but its applicability to canine HSA has not been explored. This study aimed to evaluate L-type amino acid transporter 1 (LAT1)–mediated uptake of boronophenylalanine (BPA), a ^10^B-containing compound, and the *in vitro* therapeutic potential of BNCT in canine HSA cells.

**Methods:**

Three canine HSA cell lines (JuB2, Ud6, Re21) were assessed for BPA uptake using fluorescence measurements, fluorescence imaging, and inductively coupled plasma–atomic emission spectrometry. LAT1 involvement was examined using the LAT1 inhibitor BCH. Clonogenic assays following X-ray or neutron irradiation, with or without BPA pretreatment, were performed to calculate survival fractions, dose–response curves, D10, relative biological effectiveness (RBE), and compound biological effectiveness (CBE).

**Results:**

All cell lines demonstrated BPA uptake, which was significantly reduced by BCH, confirming LAT1-mediated transport. Time-course analysis revealed cell line–dependent differences in intracellular ^10^B accumulation. Neutron irradiation yielded greater cytotoxicity than X-rays, and BPA further enhanced this effect, consistent with the ^10^B(*n*, α)^7^Li reaction. CBE values were 2.60 (JuB2), 2.31 (Ud6), and 1.25 (Re21), aligning with differences in BPA uptake.

**Conclusion:**

These findings demonstrate functional LAT1-dependent BPA uptake and BNCT-induced cytotoxicity in canine HSA cells. The results provide foundational radiobiological evidence supporting the feasibility of BNCT in canine HSA and justify further *in vivo* investigations.

## 1 Introduction

Hemangiosarcoma (HSA), also known as angiosarcoma, which is composed of neoplastic bone marrow precursor cells or endothelial cells, is one of the most commonly encountered cancers in dogs, accounting for 0.3–2% of all tumors (1). Although canine HSA can occur anywhere in the body, it is most frequently identified in the spleen, heart, liver, skin and subcutis (2). The splenic HSA is the most common splenic malignancies in dogs, representing 32–51% of all splenic tumors (2–4). Similarly, cardiac HSA that often occurs in the right atrium or the right atrial appendage is the most common type of cardiac tumor, representing 69% of all cardiac tumors (5). The biologic behavior of these visceral HSA is typically aggressive because the tumor rupture, local invasion, dissemination and distant metastasis are seen early in the course of disease. The dissemination or metastasis frequently occur in the liver, omentum, peritoneum, and lungs, and are the main cause of death (2). Although the anthracycline-based chemotherapy after surgery has been shown to improve prognosis, middle to long-term survival remains challenging in dogs with HSA. The median survival times for dogs with splenic HSA treated with surgery followed by chemotherapy in stage I (non-ruptured, non-metastatic), stage II (ruptured) and stage III (metastatic) have been reported to be only 160–345 days, 60–186 days and 23–87 days, respectively (6–12). Therefore, new treatment strategies are needed to obtain the long-term outcome for dogs with HSA.

Boron neutron capture therapy (BNCT) is a type of radiation therapy that selectively kills tumor cells through α-particles and ^7^Li-nuclei generated by the nuclear reaction between administered boron-10 (^10^B) compound, which is widely used clinically as L-p-boronophenylalanine (BPA), and thermal neutrons (13, 14). This ^10^B(n, α)^7^Li reaction occurs mainly in tumor cells because the administered BPA tends to accumulate in the cells via the L-type amino acid transporter 1 (LAT1), which is highly expressed on tumor cell membranes (15–17). Furthermore, the ranges of the two generated particles are less than 10 μm, which does not exceed the diameter of a typical cell, so their effects are almost entirely confined to the cells that have taken up the boron drug (13). These factors enable BNCT to significantly increase therapeutic efficacy while sparing damage to normal tissues. The doses prescribed to tissues in BNCT consist of two components: a non-boron dose, which includes γ-rays and radiation generated by nuclear reactions between nitrogen and hydrogen in tissues and neutrons; and a boron-dose produced by the nuclear reaction of ^10^B with thermal neutrons, as mentioned above (18). A non-boron dose can be converted to X-ray equivalent doses by multiplying the physical doses by a relative biological effectiveness (RBE) value, because the distribution of nitrogen and hydrogen are almost uniform regardless of the tissue types (19). On the other hand, a boron-dose is highly dependent on the distribution and concentration of ^10^B within the tissue (20, 21). Therefore, the conversion factor for the boron dose, known as the compound biological effectiveness (CBE) factor, is determined experimentally based on the boron compound and the target tissue (22).

Recently, the findings of a phase I clinical trial of BNCT for cutaneous angiosarcoma in humans have been reported (23). In that trial, an overall response rate of 70% was achieved, with limited adverse events including asymptomatic amylase elevation and mild skin damage. Therefore, BNCT is also expected to be an effective treatment strategy in dogs, but its efficacy has not yet been examined either experimentally or clinically. The present study aimed to investigate the feasibility of applying BNCT to canine HSA by examining LAT1 expression, intracellular ^10^B concentration, and cell survival after neutron irradiation with BPA using canine HSA cell lines. Furthermore, the CBE value was determined by analyzing cell survival after neutron irradiation with or without BPA, as well as after X-ray irradiation.

## 2 Materials and methods

### 2.1 Cell lines and culture

Three canine HSA cell lines established from three xenograft canine HSAs were selected for this study: JuB2, Ud6 and Re21 were derived from HSA tissues with the primary site of the liver, spleen, and right atrium, respectively (24, 25). These cell lines were kindly provided by Dr. Hiroki Sakai at Gifu University, who originally established them. All three cell lines were cultured in Dulbecco's Modified Eagle Medium (D-MEM; Fujifilm Wako Pure Chemical, Osaka, Japan) with 10% fetal bovine serum (FBS; Nichirei Biosciences, Tokyo, Japan). These cell lines were incubated at 37 °C in a humidified atmosphere of 5% CO_2_ and were maintained in the late exponential phase when the flask was almost confluent.

### 2.2 One-step RT-PCR

Total RNA was isolated from each cell line using the RNA extraction solution (NucleoSpin miRNA, Macherey-Nagel, Düren, Germany). RT-PCR amplification was performed using the One-step RT-PCR kit (QIAGEN, Hilden, Germany; Cat. No. 210212) according to the manufacturer's instructions. The LAT1 gene was amplified using specific primers (forward: 5′-CTGGATCGAGCTGCTCATCATC-3′; reverse: 5′-ACATCACCCTTCCCGATCTGG-3′). Primers for the housekeeping gene RPL19 (forward: 5′-CCTTCCTCAAAAAGTCTGCG-3′; reverse: 5′-GTTCTCATCGTAGGGAGC-3′) were used as an internal control (26). A mixture of 10 μl of the RT-PCR product and 2 μl of 6 × Loading Buffer (Takara Bio, Shiga, Japan) was subjected to electrophoresis in a 2% agarose gel using Tri-acetate-EDTA (TAE) as the running buffer. The DNA bands were visualized using an ImageQuant™ LAS 500 system (GE Healthcare Japan, Tokyo, Japan).

### 2.3 BPA uptake assay

The BPA uptake capacities of three HSA cell lines were assessed using the amino acid uptake assay kit (Dojindo Laboratories, Kumamoto, Japan; Cat. No. UP04-12) according to the manufacturer's instructions. Briefly, the cells were seeded on 96-well plates (1 × 10^4^ cells/well) in the fresh growth medium and incubated at 37 °C in a humidified atmosphere of 5% CO_2_ for 12 h. After removal of the growth medium, 150 μl of BPA uptake solution was added. The uptake reaction was terminated by removing the solution followed by washing with 150 μl of Hank's Balanced Salt Solution (HBSS; Thermo Fisher Scientific, Tokyo, Japan). Then, 150 μl of working solution containing the BPA-probe was added and measurement using a plate reader and fluorescence imaging were conducted. The plate reader measurements were performed using a Nivo Alpha S (PerkinElmer Japan, Kanagawa, Japan) with excitation and emission wavelengths of 360 nm and 460 nm, respectively. Fluorescence imaging was carried out using a confocal laser scanning microscope (LSM710; Carl Zeiss, Oberkochen, Germany) equipped with a DAPI filter set. To evaluate BPA uptake via LAT1, the LAT1 inhibitor BCH (2-aminobicyclo [2.2.1] heptane-2-carboxylic acid; Tocris, a Biotechne brand, UK) was used at a concentration of 1 mM in this analysis.

### 2.4 Preparation of BPA solution and boron concentration measurement

The ^10^B-enriched boron compound BPA was obtained from Interpharma Praha, a.s. (Prague, Czech Republic). BPA (0.3 g) was suspended in distilled water containing D-fructose (0.31 g; Kanto Chemical, Tokyo, Japan) and 1.52 ml of 1N NaOH (Kanto Chemical, Tokyo, Japan) to improve its solubility. The mixture was stirred until the BPA was completely dissolved (27). Subsequently, the pH was adjusted to 7.6 with 1N HCl (Kanto Chemical, Tokyo, Japan) and was sterilized using a 0.22-μm filter. The final BPA concentration was 30 mg/ml (1.44 mg ^10^B/ml).

JuB2, Ud6, and Re21 cells were seeded at a density of 1 × 10^6^ cells/dish (10-cm in diameter) and incubated for 24 h. The culture medium was then replaced with BPA solution, and the cells were exposed to BPA-containing medium for 0.5, 1, or 2 h. The BPA solution was prepared by diluting the BPA stock solution with culture medium to achieve a final ^10^B concentration of 28 ppm. After incubation, the cells were washed twice with PBS(–) (Fujifilm Wako Pure Chemical, Osaka, Japan), followed by the addition of 2 ml of 60% nitric acid (Kanto Chemical, Tokyo, Japan) to dissolve the cells overnight. The digested samples were transferred to a graduated cylinder and diluted with distilled water to a final volume of 10 ml for subsequent analysis. The ^10^B concentration in the prepared samples was measured using an inductively coupled plasma–atomic emission spectrometer (ICP-AES; ULTIMA 2, Jobin Yvon Horiba, Kyoto, Japan). Each sample was measured in triplicate, and the mean value (in ppm) was converted to the amount of boron per 1 × 10^6^ cells (ng/10^6^ cells).

### 2.5 Irradiation

The three cell lines, JuB2, Ud6, and Re21, were each divided into three groups: neutron, BNCT, and X-ray, and irradiated as described below. For each group, samples were prepared at a density of 1 × 10^5^ cells in 1.5-ml microtubes (Watson Co., Ltd., Tokyo, Japan) 8 h before irradiation. In the neutron group, samples were irradiated with thermal neutrons at a reactor power of 1 MW for 15, 30, or 45 min (neutron fluence: 7.4–23.5 × 10^11^ n/cm^2^) using the heavy-water neutron irradiation facility at Institute for Integrated Radiation and Nuclear Science, Kyoto University (KURNS). The samples were mounted on an acrylic plate and positioned within a collimator. The thermal neutron flux and accompanying γ-ray dose were measured by gold-foil activation analysis and BeO:Na thermoluminescent dosimeters (UD-170LS; Panasonic, Osaka, Japan), respectively. The total physical absorbed dose for neutron irradiation was calculated using a kerma-based dose estimation model as previously described (28). The absorbed dose (D) was expressed as the sum of the neutron dose (D_n_), gamma-ray dose (D_γ_), and boron dose (D_b_). The D_n_ component consisted of contributions from hydrogen (D_H_) and nitrogen (D_N_) reactions. The D_N_ mainly arises from the ^14^N (*n, p*) ^14^C reaction induced by thermal neutrons, whereas the D_H_ originates from neutron interactions with hydrogen atoms, primarily involving epithermal and fast neutrons. Neutron and gamma-ray flux spectra were experimentally measured, and absorbed doses were calculated by integrating these spectra with the corresponding energy-dependent kerma coefficients. The D_b_ component was estimated based on the intracellular boron concentration and the neutron flux using the kerma coefficient for the ^10^B (*n*, α) ^7^Li reaction. In the BNCT group, samples were co-incubated with 28 ppm of ^10^B from BPA for 8 h before neutron irradiation at KURNS. For dose calculation, the intracellular boron concentration was assumed to be equal to the boron concentration in the culture medium (28 ppm), as commonly applied in *in vitro* BNCT dosimetry. The irradiation conditions were identical to those used in the neutron group. In the X-ray group, samples were irradiated with 1, 2, 4, 8, or 12 Gy of X-rays at a dose rate of 250 cGy/min using a 4 MV linear accelerator (PRIMUS Mid-Energy; Siemens Healthcare, Malvern, PA, USA) at Gifu University. During irradiation, the microtubes were placed on a 5-cm-high water-equivalent phantom (Tough Water WD-4050; Kyoto Kagaku, Kyoto, Japan) to ensure full backscatter (29). A 1-cm-thick bolus (Superflab, Cat. No. 8117-1.0; Mick Radio–Nuclear Instruments, Mount Vernon, NY, USA) was placed over the microtubes to ensure dose uniformity and to increase the surface dose. For the neutron and X-ray groups, non-irradiated cells without BPA treatment were used as controls. For the BNCT group, cells treated with BPA but not exposed to neutron irradiation were used as controls to evaluate the effect of BPA alone.

### 2.6 Cell survival assay and CBE determination

Each surviving fraction of irradiated cells was evaluated by colony formation assay. After neutron or X-ray irradiation, cells from each group in the microtubes were seeded onto 6-cm dishes at 100–500 cells per dish according to the irradiation dose and then incubated for about 2 weeks until the colonies had grown to a size that was sufficient for counting. After confirming colony formation (defined as a cluster consisting of ≥ 50 cells) in the observation dishes, the cells were rinsed with PBS(–), fixed with 99.5% ethanol (Sigma–Aldrich, Tokyo, Japan) for 10 min, and stained with 50-fold-diluted Giemsa solution (MilliporeSigma, Burlington, MA, USA) for 30 min. The surviving fraction was calculated as the ratio of the number of colonies in the irradiated dishes to that in the control dishes.

The surviving fraction at each dose or neutron fluence was fitted to the linear-quadratic (LQ) model to generate cell survival curves. The LQ model is expressed by the following equations:


$$\begin{array}{l} Surviving\;fraction\;\left(LQ\;model\right)=exp\;\left(-\alpha D-\beta D^{2}\right) \end{array}$$

The fitting parameters (α and β) and goodness-of-fit (coefficient of determination, *R*^2^) were calculated using nonlinear regression analysis. Furthermore, the D_10_ (the dose required to reduce the surviving fraction to 10%) was determined for each group, with neutron fluence converted to physical dose for the neutron and BNCT groups. Finally, the conversion factors to X-ray equivalent dose for the non-boron and boron components (i.e., RBE and CBE, respectively) were calculated from the D_10_ for each group. RBE and CBE are defined by the following equations:


$$\begin{array}{l} RBE=\;\frac{D_{10}\;\left(Xray\;group\right)\;\left[Gy\right]}{D_{10}\;\left(Neutron\;group\right)\;\left[Gy\right]}\\ CBE=\;\frac{D_{10}\;\left(Xray\;group\right)-\left[D_{10}\;\left(non-boron\;dose\;in\;BNCT\;group\right)\times RBE\right]\;\left[Gy\right]}{D_{10}\;\left(boron\;dose\;in\;BNCT\;group\right)\;\left[Gy\right]} \end{array}$$

### 2.7 Statistical analyses

All data were expressed as mean ± standard deviation (SD). The fluorescence intensity measured by the plate reader and the intracellular boron concentration at different time points in each cell line were analyzed using Kruskal–Wallis tests followed by Steel–Dwass multiple comparison tests. Statistical significance was defined as *P* < 0.05. All statistical analyses were performed using JMP pro ver. 16.2.0 software (SAS Institute Inc., Cary, NC, USA).

## 3 Results

### 3.1 BPA uptake via LAT1 in canine HSA cell lines

**Figure 1:**
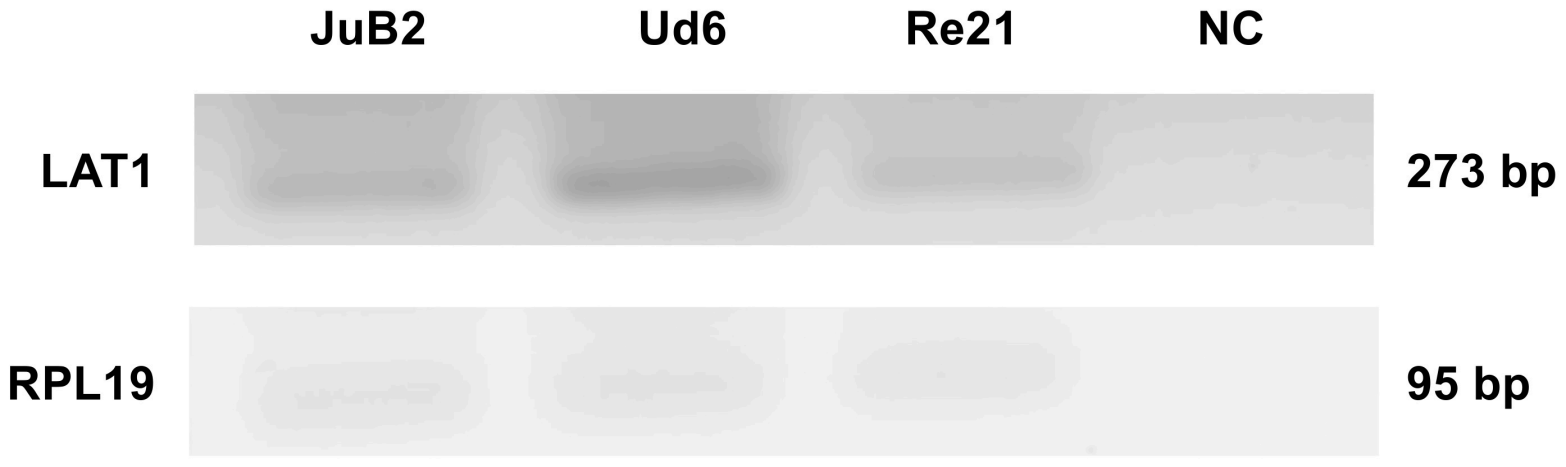
Expression of LAT1 mRNA in JuB2, Ud6, and Re21 cell lines analyzed by RT-PCR. RPL19 was used as a housekeeping gene. LAT1 amplicons (expected size: 273 bp) and RPL19 amplicons (95 bp) were detected in all cell lines, whereas no bands were observed in the negative control (NC).

RT-PCR analysis demonstrated the presence of LAT1 mRNA in all tested cell lines (Figure 1). The amplification product was of the expected size (273 bp), and amplification of the housekeeping gene RPL19 (95 bp) was also observed. No amplification was detected in the negative control.

**Figure 2:**
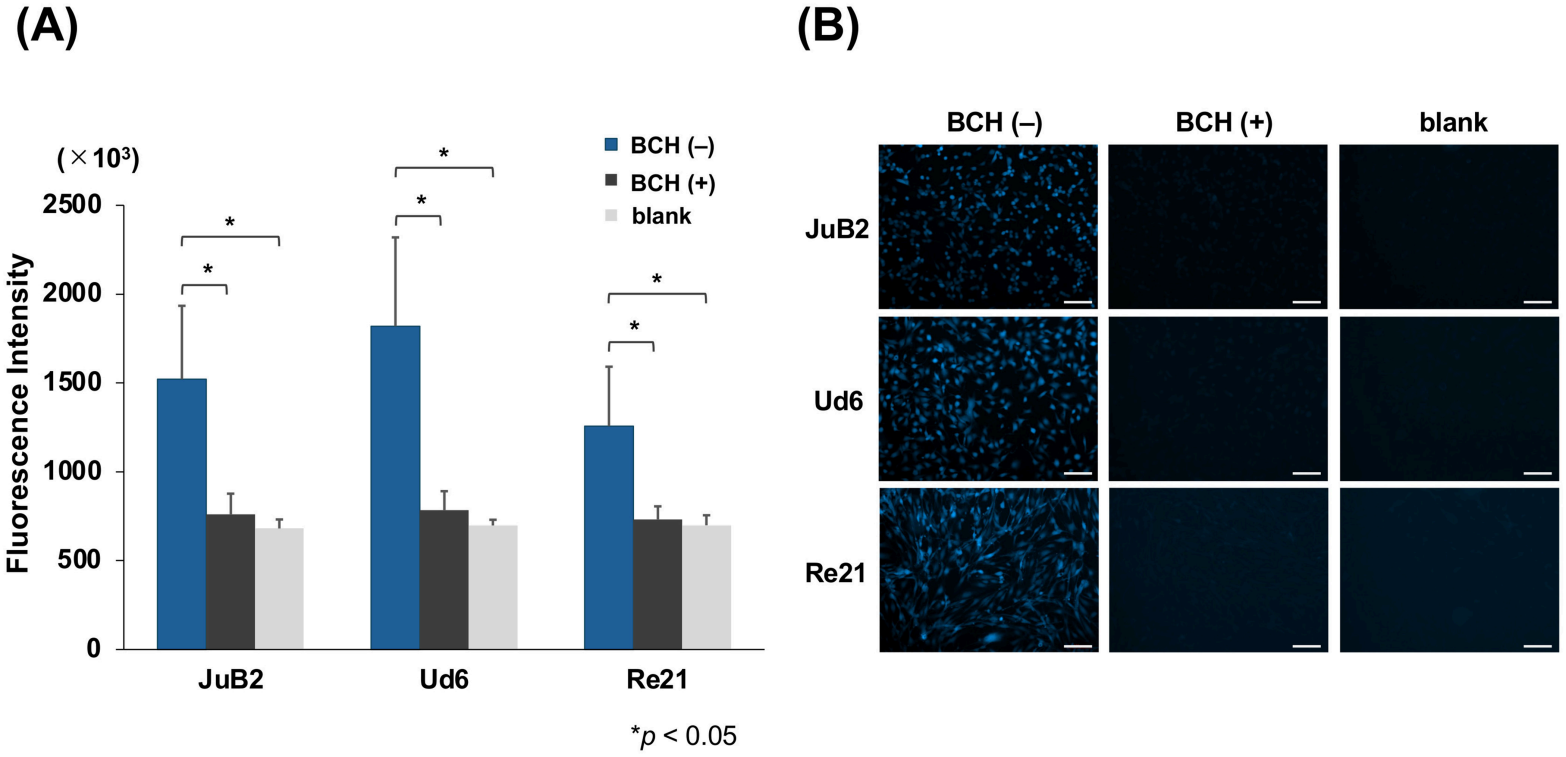
BPA uptake in JuB2, Ud6, and Re21 cell lines assessed by fluorescence intensity using plate reader measurements **(A)** and fluorescence imaging **(B)**. BPA uptake was observed in all cell lines with both methods, and the addition of the LAT1 inhibitor BCH reduced fluorescence intensity to levels comparable to the blank. Data are presented as mean ± SD. Statistical analyses were performed using Kruskal–Wallis tests followed by Steel–Dwass multiple comparison tests (*p < 0.05). Scale bar = 100 μm. Original magnification, × 40.

The cellular uptake of BPA was evaluated by measuring fluorescence intensity using a plate reader (Figure 2A). Fluorescence intensity was elevated in all cell lines relative to the blank. Among the cell lines, the intensity tended to be highest in Ud6, followed by JuB2 and Re21. Furthermore, the addition of the LAT1 inhibitor BCH reduced the fluorescence intensity in all cell lines to a level comparable to that of the blank. Similar results were obtained using fluorescence imaging (Figure 2B).

### 3.2 Intracellular ^10^B concentration

**Figure 3:**
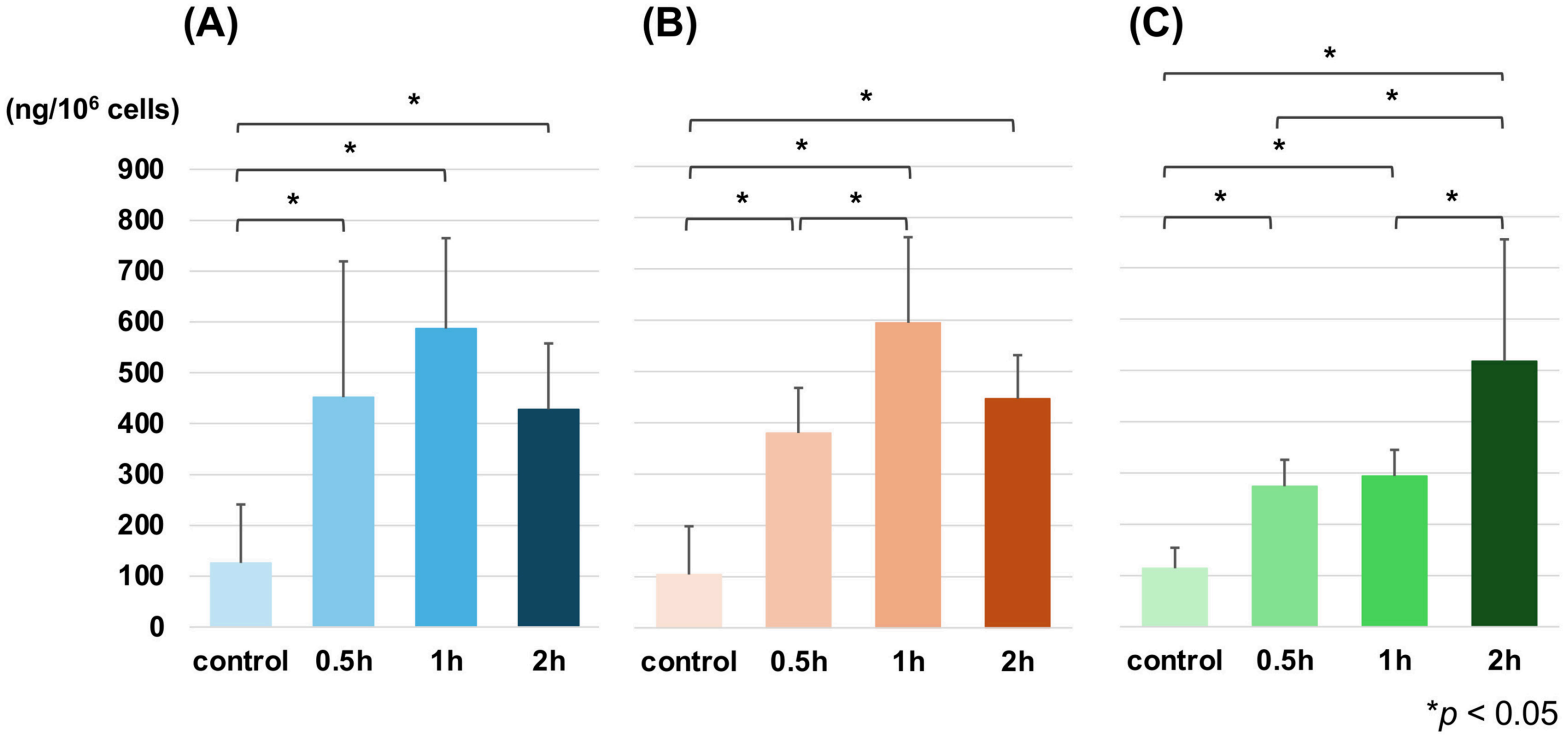
Time-dependent changes in intracellular ^10^B levels after BPA treatment in JuB2 **(A)**, Ud6 **(B)**, and Re21 **(C)** cell lines measured by ICP-AES. Cells were treated with 28 ppm BPA for up to 2 h after addition, and intracellular ^10^B levels were quantified. Data are presented as mean ± SD. Statistical analyses were performed using Kruskal–Wallis tests followed by Steel–Dwass multiple comparison tests (*p < 0.05).

Three cell lines were treated with 28 ppm of BPA, and the intracellular ^10^B levels were measured by ICP-AES up to 2 h after addition (Figure 3). In JuB2 cells, intracellular ^10^B levels were significantly increased at 0.5, 1, and 2 h following BPA treatment

compared with the control (Figure 3A). In Ud6 cells, intracellular ^10^B levels were significantly increased at 0.5, 1, and 2 h versus control, and also between 0.5 and 1 h (Figure 3B). No significant difference was observed between 1 and 2 h after addition in these two cell lines. In Re21 cells, intracellular ^10^B levels were significantly higher at 0.5, 1, and 2 h versus control, and also between 0.5 and 2 h as well as 1 and 2 h (Figure 3C). The maximum intracellular ^10^B levels were reached at 1 h in JuB2 and Ud6 cells, whereas in Re21 cells, the peak was observed at 2 h post-BPA addition. The peak intracellular ^10^B concentrations were 587.3 ± 178 ng/10^6^ cells in JuB2 cells at 1 h, 594.5 ± 167 ng/10^6^ cells in Ud6 cells at 1 h, and 518 ± 237 ng/10^6^ cells in Re21 cells at 2 h (mean ± SD, n = 3).

### 3.3 Surviving fraction after irradiation and CBE factor

**Figure 4:**
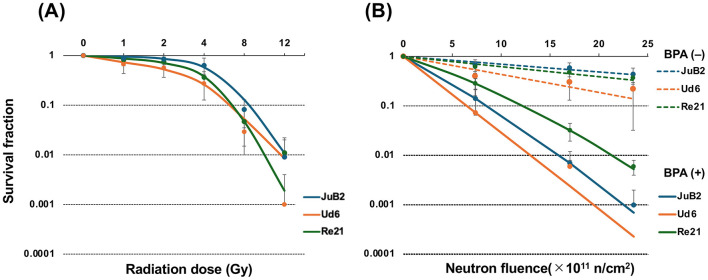
Survival fraction curves of JuB2, Ud6, and Re21 cell lines following X-ray **(A)** or neutron **(B)** irradiation. Survival fractions were determined by colony formation assays, and curves were fitted using the linear–quadratic (LQ) model. Error bars represent mean ± SD. **(A)** Survival curves after X-ray irradiation. The x-axis represents absorbed dose (Gy). **(B)** Survival curves after neutron irradiation. The x-axis represents thermal neutron fluence. Dashed lines indicate neutron irradiation without BPA (neutron group), whereas solid lines indicate neutron irradiation in the presence of BPA (BNCT group). For BNCT, cells were pre-treated with 28 ppm BPA for 8 h before neutron irradiation.

The cell survival fractions of JuB2, Ud6, and Re21 cells were determined by colony formation assays after X-ray or neutron irradiation. BPA treatment alone did not significantly affect clonogenic survival in any of the three cell lines. The obtained survival fractions were plotted on a semi-logarithmic scale, and survival curves were fitted according to the LQ model (Figure 4). In the X-ray group, the survival fraction showed little reduction at 1, 2, and 4 Gy, but decreased sharply at 8 and 12 Gy (Figure 4A). In contrast, in the neutron group, the survival fraction decreased exponentially with increasing dose, although the overall cell killing effect was relatively modest (Figure 4B). In the BNCT group, the survival fractions of all cell lines were markedly lower than those in the neutron group (Figure 4B). Among the three cell lines in the BNCT group, Ud6 exhibited the lowest survival fraction, followed by JuB2 and Re21. Survival data were fitted using the LQ model, and the resulting α and β parameters together with goodness-of-fit values are presented in Table 1.

**Table 1:** Linear–quadratic model fitting parameters for survival curves.

Cell line	Radiation type	α	β	α/β	*R* ^2^
JuB2	X-ray	0.305	0.008	38.8	0.993
	Neutron	0.139	0.003	41.42	0.988
	BNCT	0.398	0.006	69.6	1.000
Ud6	X-ray	0.016	0.030	0.521	0.989
	Neutron	0.050	0.001	65.9	0.962
	BNCT	0.246	0.003	92.2	1.000
Re21	X-ray	0.103	0.035	2.98	0.995
	Neutron	0.068	0.001	57.6	0.991
	BNCT	0.148	0.003	46.4	1.000

Subsequently, as shown in Table 2, the thermal neutron fluence was converted to the corresponding physical absorbed dose, and the D_10_ values for each cell line were determined from the cell survival curves in each irradiation group. Based on the obtained D_10_ values, the RBE and CBE were calculated (Table 3). As a result, Ud6 showed the highest RBE value of 3.47, whereas JuB2 and Re21 exhibited similar RBE values of approximately 2. The CBE values were 2.60 and 2.31 for JuB2 and Ud6, respectively, which were comparable to each other, while Re21 showed the lowest CBE value of 1.25.

**Table 2:** Dosimetry in the Neutron and BNCT group irradiated at KURNS.

Group	Irradiation time (min)	Neutron fluence ( × 10^11^ n/cm^2^)	Boron concentration in medium (ppm)	Dose component (Gy)		Total physical absorbed dose (Gy)
				Non-boron dose	Boron dose	
Neutron	15	7.35	–	0.52	–	0.52
	30	17.0	–	1.12	–	1.12
	45	23.5	–	1.63	–	1.63
BNCT	15	7.35	28	0.52	2.52	3.04
	30	17.0	28	1.12	5.39	6.51
	45	23.5	28	1.63	7.91	9.54

**Table 3:** Determination of RBE and CBE based on D_10_ in JuB2, Ud6 and Re21 cell lines.

Cell line	D_10_ in each dose component (Gy)			RBE	CBE
JuB2	X-ray		8.50	1.95	2.60
	Neutron		4.37		
	BNCT	non-boron dose	0.59		
		boron dose	2.83		
Ud6	X-ray		6.49	3.47	2.31
	Neutron		1.87		
	BNCT	non-boron dose	0.44		
		boron dose	2.14		
Re21	X-ray		6.78	2.02	1.25
	Neutron		3.35		
	BNCT	non-boron dose	0.84		
		boron dose	4.07		

## 4 Discussion

The primary objective of this study was to provide foundational *in vitro* data on the biological response of canine HSA cells to BNCT by evaluating LAT1-mediated BPA uptake and radiation-induced cytotoxicity across multiple HSA cell lines. In the present study, we demonstrated that all three canine HSA cell lines examined were capable of taking up BPA via the amino acid transporter LAT1, indicating that each cell line achieved sufficient intracellular ^10^B accumulation for BNCT. Furthermore, BNCT in the presence of BPA exerted a more pronounced cytotoxic effect compared with X-ray irradiation or neutron irradiation alone. These findings support the potential of BNCT as an effective therapeutic approach for canine HSA. Given that this tumor type frequently develops multiple metastatic lesions in organs such as the liver and lung, BNCT may warrant further investigation as a potential approach for targeting disseminated lesions, although its therapeutic applicability remains to be established *in vivo*.

One of the key findings of this study is that BPA uptake mediated by LAT1 was clearly demonstrated in canine HSA cell lines for the first time. Although increased LAT1 mRNA expression has previously been reported in canine mammary tumors, malignant melanoma, hepatocellular carcinoma and intracranial tumors (26, 30–33), its expression and functional relevance in HSA had remained unknown. In this study, BPA uptake was significantly suppressed by the LAT1 inhibitor BCH, indicating that LAT1 is functionally involved in BPA transport in these three cell lines. However, the fluorescence intensity and intracellular ^10^B levels differed among the lines, with Ud6 exhibiting the highest uptake, followed by JuB2 and Re21. Furthermore, Intracellular ^10^B levels increased rapidly after BPA addition and reached near-maximum values within 1–2 h in JuB2 and Ud6 cells, whereas Re21 cells showed a gradual increase up to 2 h. These findings indicate that substantial intracellular boron accumulation occurs within the first 1–2 h following BPA exposure, although the longer-term kinetics and retention beyond this period remain to be clarified. A previous study using the mouse squamous cell carcinoma cell line SCC7 demonstrated that variations in LAT1 expression strongly influence the amount of BPA uptake (34). Based on this evidence, the differences in fluorescence intensity and intracellular ^10^B levels observed here may reflect lower LAT1 expression in Re21 compared with JuB2 and Ud6, although LAT1 expression itself was not quantified in the present study. In JuB2 and Ud6 cells, intracellular ^10^B levels peaked 1 h after BPA addition and subsequently decreased by 2 h. This temporal pattern is likely attributable to the amino acid exchange transport mechanism of LAT1. LAT1 imports extracellular BPA in exchange for intracellular amino acids such as glutamate, while intracellular BPA can be exported in exchange for extracellular amino acids such as tyrosine (35). Therefore, in JuB2 and Ud6, intracellular BPA concentrations may have exceeded extracellular concentrations at 1 h, after which export via exchange transport predominated, resulting in decreased intracellular ^10^B levels. In contrast, Re21 may require more time for intracellular BPA to surpass extracellular levels due to relatively lower LAT1 expression, leading to a delayed peak at 2 h after BPA addition. In human angiosarcoma, high LAT1 expression has been demonstrated immunohistochemically (36, 37), and recent clinical reports have shown that BNCT can be effective in treating this malignancy (23, 38). Despite the differences in the amount and timing of BPA uptake among the cell lines, the observation that all lines exhibited substantial BPA uptake, together with supporting evidence from human angiosarcoma, suggests that LAT1-dependent BNCT may also hold promise as a therapeutic option for canine HSA.

Analysis of cell survival following X-ray and neutron irradiation revealed broadly similar patterns across the three cell lines. However, neutron irradiation produced greater cytotoxicity than X-ray irradiation, and this effect was further enhanced in the presence of BPA, indicating that the ^10^B (*n*, α) ^7^Li reaction contributed to the increased cell killing and supporting the potential effectiveness of BNCT. In the present study, the BPA concentration in the culture medium was set at 28 ppm, and intracellular boron accumulation was evaluated under this condition. For the purpose of dose estimation in the BNCT experiments, the boron concentration in the culture medium was assumed to approximate the intracellular boron concentration under these experimental conditions. In canine clinical BNCT studies, intravenous administration of BPA at 350 mg/kg over 45 min has been reported to achieve blood boron concentrations of approximately 10–12 ppm during neutron irradiation, as estimated using a two-compartment pharmacokinetic model (39). Because direct measurement of intratumoral boron concentration during irradiation is generally not feasible, tumor boron levels are commonly estimated using a tumor-to-blood ratio, often assumed to be approximately 3.0–3.5 (40, 41). Based on this ratio, the estimated intratumoral boron concentration would be approximately 30–42 ppm. Moreover, it has been generally suggested that intratumoral boron concentrations of at least 20 ppm are required to achieve effective tumor control in BNCT (42). In this context, the 28 ppm boron concentration used in the present *in vitro* experiments appears to be within a clinically relevant and therapeutically meaningful range. The degree of enhanced effect in the BNCT groups can be partially explained by the CBE values, which represent the conversion factor used to express the boron dose component as a biologically X-ray–equivalent dose. Based on the D_10_ values, the calculated CBE values were 2.60 for JuB2, 2.31 for Ud6, and 1.25 for Re21, demonstrating that Re21 benefited the least from BNCT. Although the relationships among BPA-derived fluorescence intensity, intracellular ^10^B accumulation, cell survival, and CBE values were not perfectly proportional, the overall trend was largely consistent, particularly with Re21 exhibiting lower boron accumulation and reduced BNCT responsiveness. These findings suggest that BNCT responsiveness differs among the cell lines and may be associated with variations in LAT1-mediated BPA uptake and the resulting intracellular ^10^B concentration. Previous studies have reported a wide range of CBE values depending on the tumor type, boron compound, dose, biological endpoint, and neutron beam quality. For example, experimental studies using SCC VII tumor cells have reported BPA-CBE values in the range of approximately 3–4 under specific irradiation conditions (43), while a reassessment of BPA-mediated effects in hepatocytes revealed that CBE values can vary substantially with physical dose, showing values around 4 at very low doses but decreasing toward ~1 at higher doses (44). Other reviews have emphasized that CBE differs markedly between BPA and sodium mercaptoundecahydro-dodecaborate (BSH) and should not be compared across experiments without careful consideration of beam parameters and biological context (45). Because CBE is influenced by multiple biological and physical variables, direct numerical comparisons between studies should be made with caution. Although the CBE values obtained in our study were slightly lower than those reported previously, the boron dose component still produced a sufficiently strong cytotoxic effect. However, the relatively low CBE value of Re21 may reflect its reduced LAT1-dependent uptake of BPA, leading to lower intracellular ^10^B accumulation. Verification of this hypothesis will require direct assessment of LAT1 protein expression, quantitative boron imaging, and *in vivo* BNCT evaluation to determine whether the observed *in vitro* differences translate into therapeutic outcomes.

Despite these findings, several limitations of this study should be considered. First, although LAT1 involvement in BPA transport was supported by the inhibitory effect of BCH, our study did not directly quantify LAT1 protein expression in the three cell lines. Therefore, we cannot establish a definitive correlation between LAT1 expression levels, intracellular boron accumulation, and BNCT sensitivity. Quantitative protein-level analyses, such as Western blotting, flow cytometry, or immunocytochemistry, will be necessary to clarify whether differences in LAT1 abundance account for the variability in BPA uptake and CBE values. Second, BPA uptake was evaluated only up to 2 h after exposure, leaving the longer-term kinetics and retention characteristics of BPA in canine HSA cells uncertain. Extended time-course experiments would help determine how sustained boron accumulation influences BNCT efficacy. Third, all functional and radiobiological experiments were conducted *in vitro*. Since *in vitro* systems cannot fully replicate the complexity of the *in vivo* tumor microenvironment, *in vivo* BNCT studies will be necessary to verify whether the observed effects translate into therapeutic benefit in more physiologically relevant settings. Another limitation of this study is the absence of comparative analyses between tumor cells and normal canine cells to evaluate tumor selectivity. While the present study focused on demonstrating BNCT-induced cytotoxicity in canine HSA cells as a foundational step, assessment of boron uptake and radiation sensitivity in normal canine endothelial or stromal cells will be necessary to further clarify therapeutic selectivity and safety. An additional limitation of this study is the use of monolayer cell culture models, which do not fully recapitulate the complex tumor microenvironment of canine HSA. *In vivo*, HSA tumors are characterized by abnormal vascular architecture, hypoxic regions, and cellular heterogeneity, all of which may influence boron distribution, LAT1 expression, and radiation responsiveness. For example, hypoxia may alter amino acid transporter activity and affect BPA uptake, while vascular irregularities could impact boron delivery and spatial dose distribution during neutron irradiation. Therefore, the present *in vitro* findings should be interpreted with caution, and further investigations using three-dimensional culture systems and *in vivo* models will be necessary to better reflect clinical conditions.

In conclusion, this study should be regarded as a proof-of-principle investigation demonstrating the radiobiological feasibility of BNCT in canine HSA cell lines. We demonstrated that canine HSA cell lines effectively take up BPA through LAT1-mediated transport and exhibit sensitivity to BNCT in this study. BPA uptake was confirmed by fluorescence assays and ICP-AES analysis, and the addition of BCH supported the functional involvement of LAT1 in this process. Radiation survival analyses further revealed that BNCT significantly enhanced cell-killing effects compared with X-ray or neutron irradiation alone, underscoring the contribution of the ^10^B (*n*, α) ^7^Li reaction to cytotoxicity. Collectively, these findings provide a radiobiological rationale for further preclinical investigation. However, therapeutic applicability must be validated through appropriate *in vivo* studies before clinical translation can be considered.

## Data Availability

The original contributions presented in the study are included in the article/Supplementary material, further inquiries can be directed to the corresponding author.

## Supplementary material

Supplementary Data Sheet 1 Original uncropped gel images of LAT1 and RPL19 PCR products corresponding to Figure 1. Images are provided as raw, unedited source data.

## References

[1] Clifford CA Mackin AJ Henry CJ. Treatment of canine hemangiosarcoma: 2000 and beyond. J Vet Intern Med. (2000) 14:479–85. doi: 10.1111/j.1939-1676.2000.tb02262.x 11012108

[2] Mullin C Clifford CA. Hemangiosarcoma. In:Vail DM Thamm DH Liptak JM, editors. Withrow and MacEwen's Small Animal Clinical Oncology 6th ed. St. Louis, MO: Saunders Elsevier (2020). p. 773-778.

[3] Davies O Taylor AJ. Refining the “double two-thirds” rule: Genotype-based breed grouping and clinical presentation help predict the diagnosis of canine splenic mass lesions in 288 dogs. Vet Comp Oncol. (2020) 18:548–58. doi: 10.1111/vco.12574 32043696

[4] Ziogaite B Contreras ET Horgan JE. Incidence of splenic malignancy and hemangiosarcoma in dogs undergoing splenectomy surgery at a surgical specialty clinic: 182 cases (2017-2021). PLoS ONE. (2024) 19:e0314737. doi: 10.1371/journal.pone.0314737 39625875 PMC11614263

[5] Treggiari E Pedro B Dukes-McEwan J Gelzer AR Blackwood L. A descriptive review of cardiac tumours in dogs and cats. Vet Comp Oncol. (2017) 15:273–88. doi: 10.1111/vco.12167 26420436

[6] Sorenmo KU Jeglum KA Helfand SC. Chemotherapy of canine hemangiosarcoma with doxorubicin and cyclophosphamide. J Vet Intern Med. (1993) 7:370–6. doi: 10.1111/j.1939-1676.1993.tb01033.x 8114034

[7] Kim SE Liptak JM Gall TT Monteith GJ Woods JP. Epirubicin in the adjuvant treatment of splenic hemangiosarcoma in dogs: 59 cases (1997-2004). J Am Vet Med Assoc. (2007) 231:1550–7. doi: 10.2460/javma.231.10.1550 18021000

[8] Wendelburg KM Price LL Burgess KE Lyons JA Lew FH Berg J. Survival time of dogs with splenic hemangiosarcoma treated by splenectomy with or without adjuvant chemotherapy: 208 cases (2001-2012). J Am Vet Med Assoc. (2015) 247:393–403. doi: 10.2460/javma.247.4.393 26225611

[9] Matsuyama A Poirier VJ Mantovani F Foster RA Mutsaers AJ. Adjuvant doxorubicin with or without metronomic cyclophosphamide for canine splenic hemangiosarcoma. J Am Anim Hosp Assoc. (2017) 53:304–12. doi: 10.5326/JAAHA-MS-6540 28892429

[10] Batschinski K Nobre A Vargas-Mendez E Tedardi MV Cirillo J Cestari G . Canine visceral hemangiosarcoma treated with surgery alone or surgery and doxorubicin: 37 cases (2005-2014). Can Vet J. (2018) 59:967–72. 30197439 PMC6091137

[11] Ciepluch BJ Wilson-Robles HM Pashmakova MB Budke CM Ellison GW Thieman Mankin KM. Long-term postoperative effects of administration of allogeneic blood products in 104 dogs with hemangiosarcoma. Vet Surg. (2018) 47:1039–45. doi: 10.1111/vsu.12967 30242852

[12] Marconato L Chalfon C Finotello R Polton G Vasconi ME Annoni M . Adjuvant anthracycline-based vs metronomic chemotherapy vs no medical treatment for dogs with metastatic splenic hemangiosarcoma: a multi-institutional retrospective study of the Italian Society of Veterinary Oncology. Vet Comp Oncol. (2019) 17:537–44. doi: 10.1111/vco.12519 31251441

[13] Coderre JA Morris GM. The radiation biology of boron neutron capture therapy. Radiat Res. (1999) 151:1–18. doi: 10.2307/3579742 9973079

[14] Barth RF Gupta N Kawabata S. Evaluation of sodium borocaptate (BSH) and boronophenylalanine (BPA) as boron delivery agents for neutron capture therapy (NCT) of cancer: an update and a guide for the future clinical evaluation of new boron delivery agents for NCT. Cancer Commun. (2024) 44:893–909. doi: 10.1002/cac2.12582 38973634 PMC11337926

[15] Yoshimoto M Kurihara H Honda N Kawai K Ohe K Fujii H . Predominant contribution of L-type amino acid transporter to 4-borono-2-(18)F-fluoro-phenylalanine uptake in human glioblastoma cells. Nucl Med Biol. (2013) 40:625–9. doi: 10.1016/j.nucmedbio.2013.02.010 23557719

[16] Yanagida O Kanai Y Chairoungdua A Kim DK Segawa H Nii T . Human L-type amino acid transporter 1 (LAT1): characterization of function and expression in tumor cell lines. Biochim Biophys Acta. (2001) 1514:291–302. doi: 10.1016/S0005-2736(01)00384-4 11557028

[17] Fuchs BC Bode BP. Amino acid transporters ASCT2 and LAT1 in cancer: partners in crime? Semin Cancer Biol. (2005) 15:254–66. doi: 10.1016/j.semcancer.2005.04.005 15916903

[18] Iwasaki R Yoshikawa R Umeno R Seki A Matsukawa T Takeno S . The effects of BPA-BNCT on normal bone: determination of the CBE value in mice‡. J Radiat Res. (2023) 64:795–803. doi: 10.1093/jrr/rrad054 37517393 PMC10516729

[19] White DR Griffith RV Wilson IJ. Photon, electron, proton and neutron interaction data for body tissues (ICRU Report 46). Bethesda (MD): International Commission on Radiation Units and Measurements; 1992.

[20] Barth RF Soloway AH Brugger RM. Boron neutron capture therapy of brain tumors: past history, current status, and future potential. Cancer Invest. (1996) 14:534–50. doi: 10.3109/07357909609076899 8951358

[21] Evangelista L Jori G Martini D Sotti G. Boron neutron capture therapy and 18F-labelled borophenylalanine positron emission tomography: a critical and clinical overview of the literature. Appl Radiat Isot. (2013) 74:91–101. doi: 10.1016/j.apradiso.2013.01.001 23395785

[22] Morris GM Coderre JA Hopewell JW Micca PL Rezvani M. Response of rat skin to boron neutron capture therapy with p-boronophenylalanine or borocaptate sodium. Radiother Oncol. (1994) 32:144–53. doi: 10.1016/0167-8140(94)90101-5 7972908

[23] Kashihara T Nakamura S Yamazaki N Takahashi A Namikawa K Ogata D . Boron neutron capture therapy for cutaneous angiosarcoma and malignant melanoma: First in-human phase I clinical trial. Radiother Oncol. (2025) 202:110607. doi: 10.1016/j.radonc.2024.110607 39489429

[24] Kodama A Sakai H Matsuura S Murakami M Murai A Mori T . Establishment of canine hemangiosarcoma xenograft models expressing endothelial growth factors, their receptors, and angiogenesis-associated homeobox genes. BMC Cancer. (2009) 9:363. doi: 10.1186/1471-2407-9-363 19825192 PMC2768746

[25] Murai A Asa SA Kodama A Hirata A Yanai T Sakai H. Constitutive phosphorylation of the mTORC2/Akt/4E-BP1 pathway in newly derived canine hemangiosarcoma cell lines. BMC Vet Res. (2012) 8:128. doi: 10.1186/1746-6148-8-128 22839755 PMC3438112

[26] Fukumoto S Hanazono K Komatsu T Ueno H Kadosawa T Iwano H . L-type amino acid transporter 1 (LAT1): a new therapeutic target for canine mammary gland tumour. Vet J. (2013) 198:164–9. doi: 10.1016/j.tvjl.2013.06.016 23896327

[27] Yoshino K Suzuki A Mori Y Kakihana H Honda C Mishima Y . Improvement of solubility of p-boronophenylalanine by complex formation with monosaccharides. Strahlenther Onkol. (1989) 165:127–9. 2928932

[28] Niimi A Yoshihashi S Kawai N Yamazaki A Uritani A Iwasaki R . Evaluation of Boron Neutron Capture Therapy for treating canine oral malignant melanoma. Appl Radiat Isot. (2025) 225:112081. doi: 10.1016/j.apradiso.2025.112081 40779850

[29] Fitzpatrick CL Farese JP Milner RJ Salute ME Rajon DA Morris CG . Intrinsic radiosensitivity and repair of sublethal radiation-induced damage in canine osteosarcoma cell lines. Am J Vet Res. (2008) 69:1197–202. doi: 10.2460/ajvr.69.9.1197 18764694

[30] Fukumoto S Hanazono K Fu DR Endo Y Kadosawa T Iwano H . A new treatment for human malignant melanoma targeting L-type amino acid transporter 1 (LAT1): a pilot study in a canine model. Biochem Biophys Res Commun. (2013) 439:103–8. doi: 10.1016/j.bbrc.2013.08.020 23954667

[31] Fukumoto S Hanazono K Komatsu T Iwano H Kadosawa T Uchide T. L-type amino acid transporter 1 (LAT1) expression in canine mammary gland tumors. J Vet Med Sci. (2013) 75:431–7. doi: 10.1292/jvms.12-0356 23171689

[32] Ogihara K Naya Y Sato R Onda K Ochiai H. Analysis of L-type amino acid transporter in canine hepatocellular carcinoma. J Vet Med Sci. (2015) 77:527–34. doi: 10.1292/jvms.14-0392 25649314 PMC4478731

[33] Utsugi S Ogihara K Naya Y Sunden Y Nakamoto Y Okamoto Y. Expression of L-type amino acid transporter 1 in canine and feline intracranial tumors. J Vet Med Sci. (2022) 84:1111–7. doi: 10.1292/jvms.21-0646 35753782 PMC9412071

[34] Watanabe T Sanada Y Hattori Y Suzuki M. Correlation between the expression of LAT1 in cancer cells and the potential efficacy of boron neutron capture therapy. J Radiat Res. (2023) 64:91–8. doi: 10.1093/jrr/rrac077 36371738 PMC9855323

[35] Nomoto T Yao Y Inoue Y Suzuki M Kanamori K Takemoto H . Fructose-functionalized polymers to enhance therapeutic potential of p-boronophenylalanine for neutron capture therapy. J Control Release. (2021) 332:184–93. doi: 10.1016/j.jconrel.2021.02.021 33636247

[36] Koshi H Sano T Handa T Yanagawa T Saitou K Nagamori S . L-type amino acid transporter-1 and CD98 expression in bone and soft tissue tumors. Pathol Int. (2015) 65:460–7. doi: 10.1111/pin.12323 26134029

[37] Shimizu A Kaira K Okubo Y Utsumi D Yasuda M Tominaga H . Prognostic impact of LAT1 and CD98 expression in cutaneous angiosarcoma. Neoplasma. (2017) 64:283–8. doi: 10.4149/neo_2017_216 28052681

[38] Igaki H Murakami N Nakamura S Yamazaki N Kashihara T Takahashi A . Scalp angiosarcoma treated with linear accelerator-based boron neutron capture therapy: a report of two patients. Clin Transl Radiat Oncol. (2022) 33:128–33. doi: 10.1016/j.ctro.2022.02.006 35252597 PMC8892501

[39] Schwint AE Monti Hughes A Garabalino MA Santa Cruz GA González SJ Longhino J . Clinical Veterinary Boron Neutron Capture Therapy (BNCT) Studies in Dogs with Head and Neck Cancer: Bridging the Gap between Translational and Clinical Studies. Biology. (2020) 9:327. doi: 10.3390/biology9100327 33036386 PMC7599538

[40] Nakai K Kumada H Yamamoto T Tsurubuchi T Zaboronok A Matsumura A. Feasibility of boron neutron capture therapy for malignant spinal tumors. Appl Radiat Isot. (2009) 67:S43–6. doi: 10.1016/j.apradiso.2009.03.089 19376723

[41] Yanagie H Kumada H Sakurai Y Nakamura T Furuya Y Sugiyama H . Dosimetric evaluation of neutron capture therapy for local advanced breast cancer. Appl Radiat Isot. (2009) 67:S63–6. doi: 10.1016/j.apradiso.2009.03.110 19427224

[42] Barth RF Zhang Z Liu T. A realistic appraisal of boron neutron capture therapy as a cancer treatment modality. Cancer Commun. (2018) 38:36. doi: 10.1186/s40880-018-0280-5 29914575 PMC6006699

[43] Masunaga S Sakurai Y Tanaka H Tano K Suzuki M Kondo N . The dependency of compound biological effectiveness factors on the type and the concentration of administered neutron capture agents in boron neutron capture therapy. Springerplus. (2014) 3:128. doi: 10.1186/2193-1801-3-128 25674433 PMC4320213

[44] Ono K Tanaka H Suzuki M. Reevaluation of CBE value of BPA for hepatocytes. Appl Radiat Isot. (2020) 161:109159. doi: 10.1016/j.apradiso.2020.109159 32250845

[45] Fukuda H. Response of Normal Tissues to Boron Neutron Capture Therapy (BNCT) with ^10^B-Borocaptate Sodium (BSH) and ^10^B-Paraboronophenylalanine (BPA). Cells. (2021) 10:2883. doi: 10.3390/cells10112883 34831105 PMC8616460

