# Supplementary figures and images for: The boron uptake via LAT1 and survival analysis after boron neutron capture therapy in canine hemangiosarcoma cell lines
Source: Front Vet Sci. 2026 May 12;13:1763965. doi: 10.3389/fvets.2026.1763965 (PMC13201519; doi:10.3389/fvets.2026.1763965)

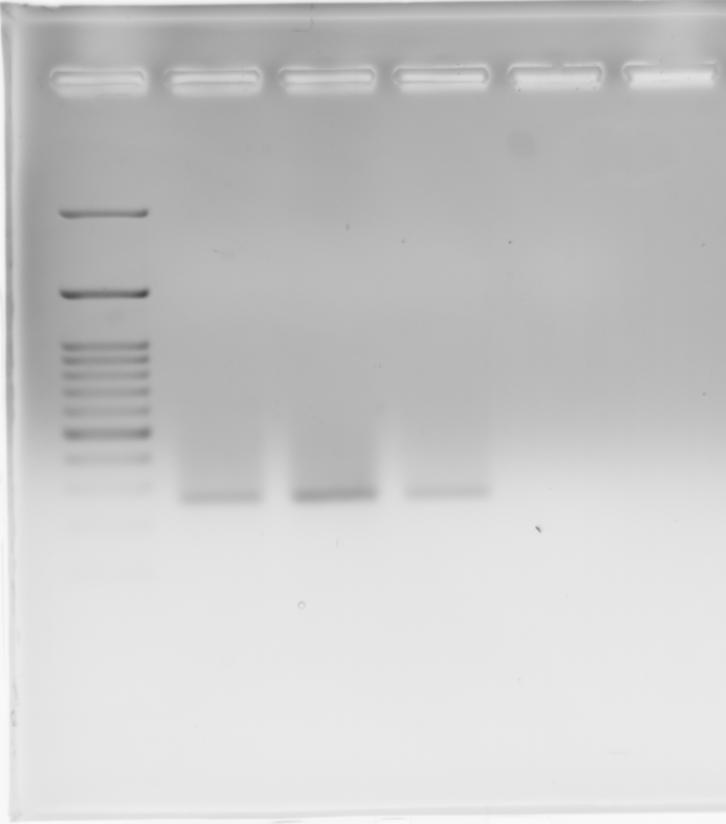

Supplement: Supplementary Data Sheet 1 — Original uncropped gel images of LAT1 and RPL19 PCR products corresponding to Figure 1. Images are provided as raw, unedited source data. [file Data_Sheet_1.zip › original images/LAT1.tif]

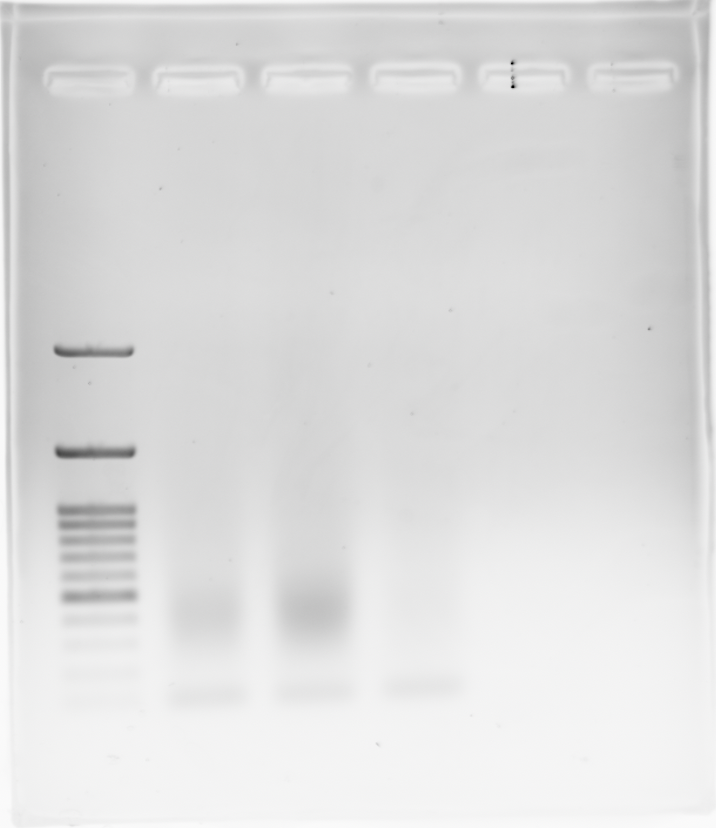

Supplement: Supplementary Data Sheet 1 — Original uncropped gel images of LAT1 and RPL19 PCR products corresponding to Figure 1. Images are provided as raw, unedited source data. [file Data_Sheet_1.zip › original images/PRL19.tif]
